# Characterization of Perineum Elasticity and Pubic Bone-Perineal Critical Distance with a Novel Tactile Probe: Results of an Intraobserver Reproducibility Study

**DOI:** 10.4236/ojog.2020.1040044

**Published:** 2020-04

**Authors:** Justin S. Brandt, Todd Rosen, Heather Van Raalte, Viktors Kurtenos, Vladimir Egorov

**Affiliations:** 1 Department of Obstetrics, Gynecology, and Reproductive Sciences, Division of Maternal-Fetal Medicine, Rutgers Robert Wood Johnson Medical School, New Brunswick, NJ, USA; 2 Princeton Urogynecology, Princeton, NJ, USA; 3 Advanced Tactile Imaging, Inc., Trenton, NJ, USA

**Keywords:** Perineal Elasticity, Tactile Imaging, Elastography, Antepartum Predictive Model, Delivery Mode, Perineum, Maternal Birth Trauma

## Abstract

**Background:**

Tactile imaging provides biomechanical mapping of soft tissues. Objective biomechanical and anatomical assessment of critical structures within the vagina and pelvis may allow development and validation of a clinical tool that could assist with clinical decisions regarding obstetrical procedures and mode of delivery. Objective: To assess intraobserver reproducibility of measurements of perineal elasticity and pubic bone-perineal critical distance with a novel tactile probe in pregnant women.

**Methods:**

An Antepartum Tactile Imager (ATI) was designed with a vaginal probe resembling a fetal skull. The probe comprises 128 tactile sensors on a double curved surface and measures 46 mm in width and 72 mm in length. The probe has a motion tracking sensor that allows acquisition of 3D tactile images. There were two arms of the study. In the first arm, biomechanical mapping of the perineum and pelvic bone location was performed in 10 non-pregnant women for purposes of demonstrating safety and feasibility. In the second arm, biomechanical mapping was performed in 10 pregnant women to explore intraobserver reproducibility. Each subject had two standardized examinations over 3 – 5 minutes by the same observer. Examination comfort and pain levels were assessed by post-procedure survey. Reproducibility was analyzed by intraclass correlation coefficients (ICC) with 95% confidence intervals and Bland-Altman plots. Bias and the 95% limits of agreement were also calculated.

**Results:**

The safety and feasibility arm of the study demonstrated high degree of safety and tolerability and reliable acquisition of tactile signals. In the reproducibility arm, 10 pregnant women were recruited at mean gestational age of 34.2 ± 6.5 weeks. The mean perineum elasticity (Young’s modulus, E) was 9.8 ± 5.9 kPa, and the mean pubic bone-perineal critical distance (D) at 20 kPa load was 34.6 ± 6.2 mm. The ICC was 0.97 [95% confidence interval (CI) 0.91, 0.99] and 0.82 [CI 0.44, 0.95] for E and D respectively, consistent with excellent intrarater agreement. The bias and the 95% limits of agreement of E were −6.3% and −29.4% to +16.7%, respectively. The bias and the 95% limits of agreement of D were −2.6% and −25.3% to +20.2%, respectively.

**Conclusions:**

The tactile imaging data obtained in the study reproducibly characterized perineal elasticity and pubic bone-perineal critical distance. Further evaluation of this tool in clinical settings is warranted.

## 1. Introduction

Dramatic changes in the mechanical properties of pelvic tissues take place antepartum to facilitate fetal delivery [1] [2] [3] [4]. Despite this, over 85% of women suffer obstetric trauma to one or several components of the pelvic floor, including the vagina, perineal body, levator ani muscles, and the anal sphincter complex [5]. Birth injury to these integral structural components leads to pelvic pain and dyspareunia and serves as a potentially modifiable risk factor for subsequent pelvic floor disorders [6] [7]. Unfortunately, there are no effective strategies to prevent perineal damage at the time of delivery beyond elective cesarean delivery, which is associated with potential short-term risks and long-term consequences [8] [9] [10] [11].

Human and animal studies show that several adaptations during pregnancy may reduce potential injury, including increased laxity of pelvic connective tissues and ligaments, increase in genital hiatal area, and alterations in the pelvic floor muscles [1] [2] [3]. Computational models of human parturition demonstrate that mechanical changes in one pelvic component may impact the susceptibility to injury of other pelvic structures [12]. However, the protective effect of pregnancy-induced alterations is variable. Therefore, quantification and knowledge of biomechanical tissue properties of critical pelvic components may allow practical prediction of behavior under applied stress during delivery and obstetrical procedures.

A new device, the Antepartum Tactile Imager (ATI), was designed to characterize perineal elasticity and pubic bone-perineal critical distance. The objective of this study was to assess safety, feasibility, and reproducibility of tactile measurements using this device. In this two-armed study, the feasibility and safety of the ATI was assessed with a cohort of non-pregnant women. Subsequently, an intraobserver reproducibility study was performed in 10 pregnant women.

## 2. Materials and Methods

### Study Design

This study was performed from March 2019 to January 2020. The safety arm was performed in non-pregnant women from March to June 2019, and the reproducibility arm was performed in pregnant women from December 2019 to January 2020. This observational study (clinical trials identifiers NCT03883867) was approved by the Western Institutional Review Board (#20183400) and the Institutional Review Board of Rutgers Robert Wood Johnson Medical School (#Pro2018002747). Written informed consent was obtained for each patient prior to study enrollment.

### Safety and Feasibility Arm

The study arm was performed in non-pregnant women as required by the Code of Federal Regulations, Title 45, §46.204(a). The arm included 10 non-pregnant women who were recruited at a private urogynecology office. Inclusion criteria were adult women, age 21+ years, with negative urine pregnancy tests and at least one prior vaginal delivery. Exclusion criteria were prior perineal surgery; HIV or hepatitis B positive serology; warty lesions on the vulva; extensive varicose veins on the vulva; active skin infection or ulceration within the vagina/vulva (e.g. Herpes infection); presence of a vaginal septum; and severe hemorrhoids.

During the examination, patients were placed in the dorsal lithotomy position. The ATI probe was covered by a disposable plastic sheath with a water based lubricant. The probe was inserted in the vagina until the sensing surface was internalized, approximately 55 mm. The probe was pushed downward to a maximum pressure load up to 20 kPa. Three orthogonal projections of the 3D vaginal pressure map with real-time ATI probe location was observed by the operator. The complete examination lasted 3 – 5 minutes.

Patients were queried about pain and comfort after the examination. Subjects were assessed about pain using a 4-point Likert scale with 1 = no pain, 2 = mild pain, 3 = moderate pain, and 4 = severe pain. The comfort level was assessed using a second 3-point Likert scale with 1 = more comfortable than manual palpation, 2 = as comfortable as manual palpation, and 3 = less comfortable than manual palpation.

### Reproducibility Arm

The reproducibility arm included 10 pregnant women who were approached at a regional perinatal center. Inclusion criteria were adult women, age 21+ years, who completed 35+ weeks’ gestation, had at least one vaginal delivery, and were planned for a trial of labor. Exclusion criteria were the same in the safety arm of the study. In addition, subjects were excluded in cases of a fetal demise or congenital abnormalities of the fetus.

The tactile examination and the pain and comfort assessments were the same as described above. Demographic data, including participants’ age, parity, weight, and gestational age were collected. Delivery data, including mode of delivery, perineal laceration, and neonatal birth weight were also collected.

### Antepartum Tactile Imager (ATI)

**Figure 1.**
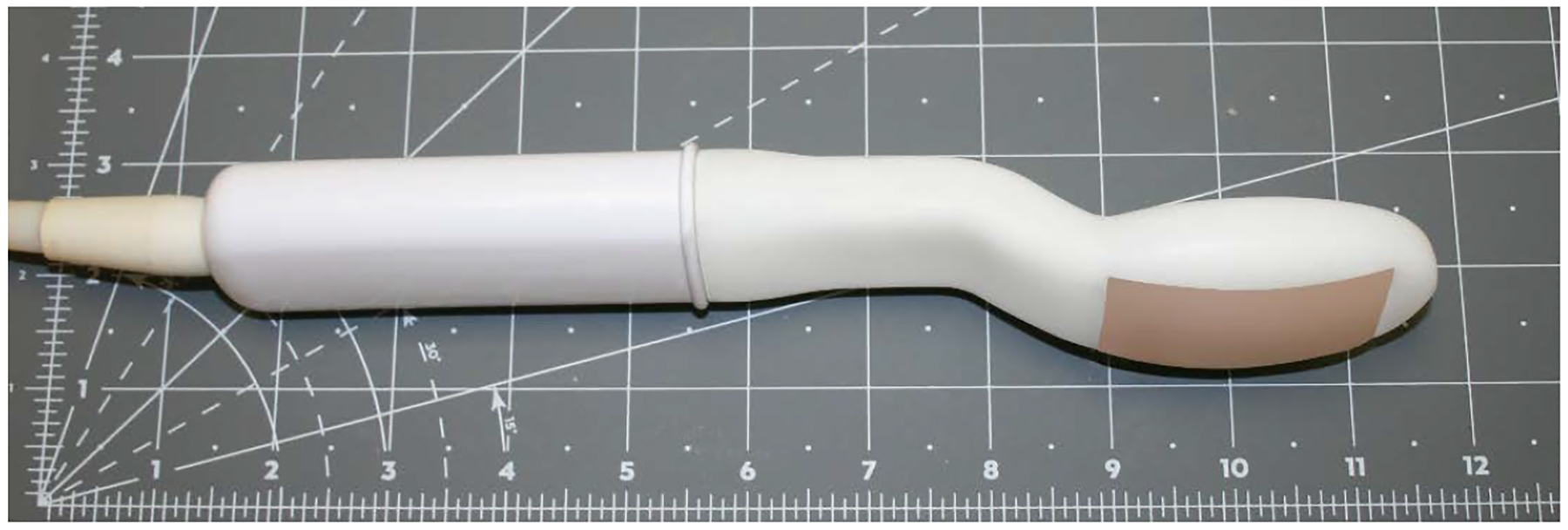
Antepartum tactile imaging probe.

The ATI was designed as a cart-based device with a medical grade touchscreen computer (Tangent, CA) and a detachable vaginal probe (Figure 1). The ATI probe contains a tactile array with 128 sensors on a double curved surface resembling a fetal skull. The probe’s dimensions are 46 mm in width and 72 mm in length, curvature radius of 60 mm in transverse and 102 mm in sagittal cross-sections, a rectangular sensing area of 26 mm by 55 mm. The probe has a 6 degree-of-freedom electromagnetic motion tracking sensor (Polhemus, Inc., VT), which allows acquisition of 3D tactile images. The referenced motion sensor was placed under the seat of a custom designed examination chair to eliminate interference for electromagnetic tracking. Biomechanical mapping of the pelvic tissues/structures was performed with attention to the perineum and pubic symphysis location.

Proprietary data processing software was used to calculate perineal elasticity and anatomical distance from the pubic bone and perineal surface, termed the pubic bone-perineal critical distance, at 20 kPa load by the ATI probe. The ATI has quantified perineal elasticity using Young’s modulus, which is calculated from spatial gradients in the resulting 3D tactile images with the use of a non-linear model for the tissue [13]. This approach was validated with multiple pelvic floor models built with two-component silicone (GE Silicones, NY) [14].

### Statistical Analysis

Descriptive statistical calculations were performed to ascertain the mean and standard deviations for perineum elasticity (E) and pubic bone-perineal critical distance (D). Intraclass correlation coefficients (ICC) were calculated for these measurements with 95% confidence intervals (CI) [15]. In addition, the following parameters were calculated as described by Bland and Altman [16]: 1) bias [*i.e*., the mean of the proportionate difference (the difference between two elasticity measurements divided by the average value of two measurements)]; and 2) 95% limits of agreement (*i.e*., 1.96 times the standard deviation of the mean of the proportionate difference). Statistical analysis was performed with STATA version 10.1 (StataCorp LP, TX) and MATLAB version R2018a (MathWorks, MA).

## 3. Results

### Feasibility and Safety Arm

The study with 10 non-pregnant women demonstrated reliable acquisition of tactile signals and composition of 3D tactile images. The imaging post-processing allowed calculation of E and D for each case. The mean E was 10.4 ± 2.5 kPa, and the mean D was 19.6 ± 6.1 mm. The pain and comfort assessments revealed a high degree of tolerability. Patients reported that the vaginal tactile imaging was minimally painful (rated 1.7 ± 0.8) and similar to manual palpation (rated 2.2 ± 0.8). No adverse events were reported.

### Reproducibly Arm

10 pregnant women were recruited for the reproducibility arm. ATI measurements were performed at a mean gestational age of 36.8 ± 0.6 weeks. All patients were multiparous and met the study’s inclusion criteria. Demographic characteristics and birth outcomes are listed in Table 1. No adverse events were reported.

**Figure 2.**
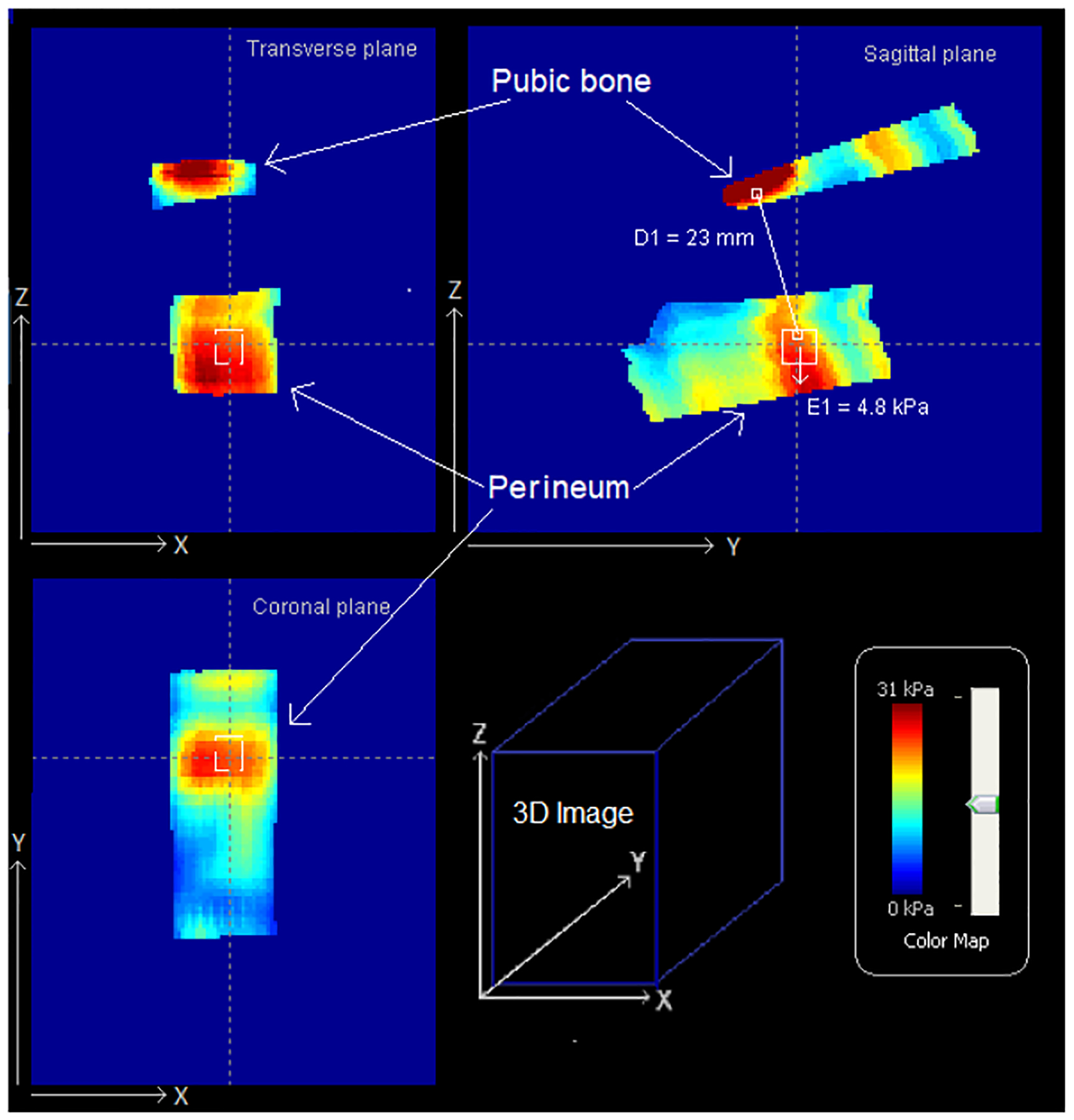
Three-dimensional tactile image of perineum elasticity and pubic bone-perineal critical distance. Three-dimensional tactile image acquired for a 34-year-old women at 37 weeks’ gestation. Attention was given to perineal elasticity and pubic bone-perineal critical distance at a 20 kPa load.

The mean E was 9.8 ± 5.9 kPa, and the mean D was 34.6 ± 6.2 mm. Figure 2 illustrates the 3 orthogonal projections of the 3D vaginal pressure map with real-time ATI probe location that was captured by the observer in case #6, which was accompanied by a second degree perineal laceration at vaginal delivery.

**Figure 3.**
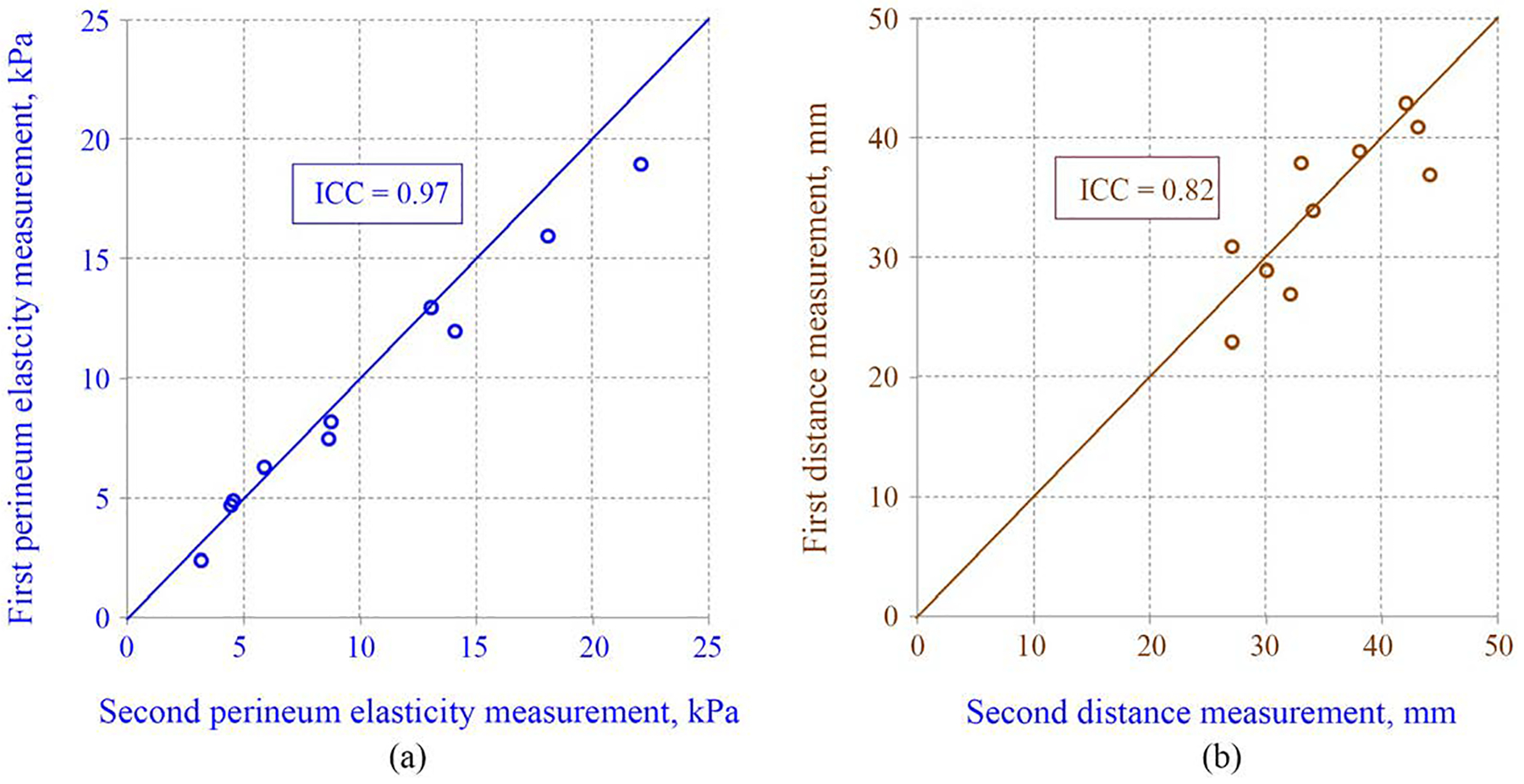
Intraclass correlation coefficients. Intraclass correlation coefficients (ICC) for two measurements of perineum elasticity (a) and pubic bone-perineal surface distance (b) by the same operator.

**Figure 4.**
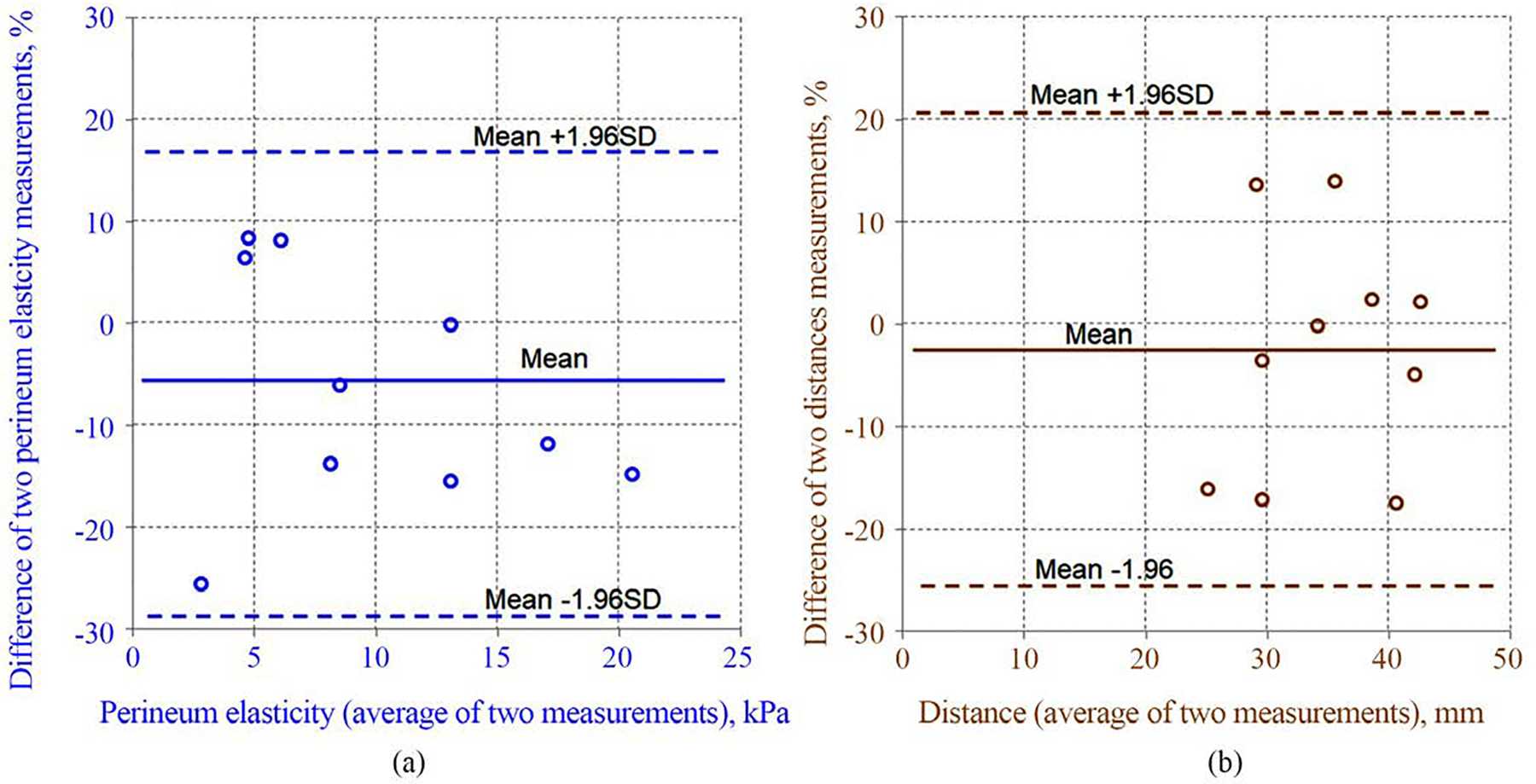
Bland-Altman scatter plots of difference between two measurements. Bland-Altman scatter plot of the percentage difference between two measurements of perineum elasticity (a) and pubic bone-perineal critical distance (b) by the same operator. The solid lines represent the proportionate mean difference; the dashed lines represent the 95% limits of agreement.

The ICC for E was 0.97 [95% CI 0.91, 0.99], which indicates excellent intrarater agreement. The bias and the 95% limits of agreement of E measurement are −6.3% and −29.4% to +16.7%. The ICC for D measurement was 0.82 [95% CI 0.44, 0.95], which also indicates excellent agreement. The bias and 95% limits of agreement of the D are −2.6% and −25.3% to +20.2%. These results are illustrated graphically in Figure 3 and Figure 4.

The pain and comfort assessments revealed a high degree of tolerability. Patients reported that the vaginal tactile imaging was minimally painful (rated 1.7 ± 0.7) and similar to manual palpation (rated 1.9 ± 0.7).

## 4. Discussion

### Principal Findings

In this study, we assessed the feasibility, safety, and reproducibility of the ATI, a novel vaginal tactile imager, in a cohort of 10 non-pregnant women and 10 pregnant women. In the feasibility and safety arm, the ATI demonstrated reliable acquisition of tactile signals and composition of 3D tactile images and patients reported pain and comfort assessments consistent with a high degree of tolerability. In the reproducibility arm, the ATI characterized perineum elasticity and pubic bone-perineal critical distance with excellent intrarater agreement.

### Results of the Study in Related Context

In general, elasticity imaging is based on generating stress in the studied tissue *in vivo* using various static or dynamic means and assessing the resulting strain by ultrasound, MRI [17]–[27] or measurement through tactile imaging [28] [29] [30]. At present, there is no ultrasound probe capable of stress-strain quantitative assessment of the entire birth canal. The ultrasound strain, as well as shear wave elastography, can measure the elasticity in only specifically defined local regions at a time. The results of the current research demonstrate that tactile imaging allows acquisition of 3D stress-strain data and elasticity assessment of the perineum.

Despite the obvious fact that vaginal delivery is a biomechanical process, currently there are no tools in clinical practice that can measure the biomechanical properties of pelvic tissue in women before delivery [31] [32] [33] [34]. We previously reported our experience with the Vaginal Tactile Imager (VTI), which was developed as a biomechanical mapping device to assess vaginal and pelvic floor conditions [30]. The VTI vaginal probe has an elongated linear design with a 96 linear sensor array on both sides of the probe [35] to allow acquisition of pressure patterns along the vaginal walls, but it does not have a 3D motion tracking system and has. The ATI has 3D imaging capability and double convex head with 2D tactile sensor array to provide stress distribution to the perineum during the ATI measurement similar to the stress distribution during the delivery.

Multiple computational models of vaginal birth have been developed [3] [12] [36] [37] [38] [39] [40]. While these models provide insight regarding the biomechanics of human parturition, they are limited by numerous assumptions, as mechanical tissue properties in late-pregnancy remain unknown. These models are often based on the data collected with cadaver tissues with the lack of muscle and tissue tone that, as a result, represent a potential source of significant inaccuracy in modeling *in vivo* deformation [41] [42] [43]. Some of these models used simplified boundary conditions and did not always consider the mechanical interaction between the fetal head and maternal pelvic tissues. Thus, to date, we cannot find a validated *in vivo* biomechanical model of vaginal delivery which could predict personalized delivery outcomes. The results of this study (and future planned exploration) may provide some of that missing data to be used in these computational models.

### Research and Clinical Implications

This study is in the first exploratory stage of the project. A finite element model development and a validation study will follow to establish nomograms for perineum elasticity and pubic bone-perineal critical distance and to establish clinically significant cut-points to guide clinical care and decision making. This new system may open a new technical capability in women’s health and change the established clinical practice. This approach may offer reliable causations between pelvic floor conditions, child size/weight on one side and delivery modes/delivery procedures and involved risk of injuries and their consequences on the other side.

### Strengths and Limitations

The strength of this study lies in the novel approach for perineum characterization. The perineal elasticity receives quantification in terms of Young’s modulus from stress-strain data. Additionally, pubic bone-perineal critical distance was quantified. This distance predicts capability of the delivery canal to pass through the fetus at predefined level of strain. Both of these measures may be associated with perineal lacerations, and the results of our study justify further prospective evaluation in clinical practice.

The study has some limitations. While the study evaluated the intrarater agreement of ATI measurements, interrater reproducibility was not evaluated. This will be performed in future studies of the ATI. In addition, the study had a small sample size with limited power to evaluate the association between perineum elasticity and pubic bone-perineal critical distance on perineal lacerations. The intention of this study, however, was not to assess clinical outcomes, but rather to assess safety, feasibility, and reproducibility of the ATI rather than efficacy.

## 5. Conclusion

In this study, the safety, feasibility, and reproducibility of a novel device were evaluated. The results of this study demonstrated that the new vaginal probe was well tolerated by study participants and produced reproducible characterization of perineum elasticity and pubic bone-perineal critical distance, both factors that may be associated with perineal lacerations and pelvic floor injury. Further evaluation of the ATI in clinical settings is warranted.

## Figures and Tables

**Table 1.** Demographic variables and delivery data for 10 pregnant women in the reproducibility arm.

Variable	N = 10
Age, years	33.5 ± 5.3
Gravida	3.7 ± 1.3
Para	1.3 ± 0.5
Gestational age at exam, weeks	36.8 ± 0.6
Intact perineum	6 (60)
Laceration	
First	3 (30)
Second	1 (10)
Third/Fourth	0
Birthweight, grams	3274 ± 276

Data presented as mean ± standard deviation and N (percent).
